# Real-Time Magnetic Resonance Imaging (MRI) during Active Wrist Motion—Initial Observations

**DOI:** 10.1371/journal.pone.0084004

**Published:** 2013-12-31

**Authors:** Robert D. Boutin, Michael H. Buonocore, Igor Immerman, Zachary Ashwell, Gerald J. Sonico, Robert M. Szabo, Abhijit J. Chaudhari

**Affiliations:** 1 Department of Radiology, University of California Davis School of Medicine, Sacramento, California, United States of America; 2 University of California Davis Imaging Research Center, Sacramento, California, United States of America; 3 Department of Orthopedic Surgery, University of California Davis School of Medicine, Sacramento, California, United States of America; The University of Queensland, Australia

## Abstract

**Background:**

Non-invasive imaging techniques such as magnetic resonance imaging (MRI) provide the ability to evaluate the complex anatomy of bone and soft tissues of the wrist without the use of ionizing radiation. Dynamic instability of wrist – occurring during joint motion – is a complex condition that has assumed increased importance in musculoskeletal medicine. The objective of this study was to develop an MRI protocol for evaluating the wrist during continuous active motion, to show that dynamic imaging of the wrist is realizable, and to demonstrate that the resulting anatomical images enable the measurement of metrics commonly evaluated for dynamic wrist instability.

**Methods:**

A 3-Tesla “active-MRI” protocol was developed using a bSSFP sequence with 475 ms temporal resolution for continuous imaging of the moving wrist. Fifteen wrists of 10 asymptomatic volunteers were scanned during active supination/pronation, radial/ulnar deviation, “clenched-fist”, and volarflexion/dorsiflexion maneuvers. Two physicians evaluated distal radioulnar joint (DRUJ) congruity, extensor carpi ulnaris (ECU) tendon translation, the scapholunate (SL) interval, and the SL, radiolunate (RL) and capitolunate (CL) angles from the resulting images.

**Results:**

The mean DRUJ subluxation ratio was 0.04 in supination, 0.10 in neutral, and 0.14 in pronation. The ECU tendon was subluxated or translated out of its groove in 3 wrists in pronation, 9 wrists in neutral, and 11 wrists in supination. The mean SL interval was 1.43 mm for neutral, ulnar deviation, radial deviation positions, and increased to 1.64 mm during the clenched-fist maneuver. Measurement of SL, RL and CL angles in neutral and dorsiflexion was also accomplished.

**Conclusion:**

This study demonstrates the initial performance of active-MRI, which may be useful in the investigation of dynamic wrist instability *in vivo.*

## Introduction

The wrist is considered unstable clinically if it exhibits symptomatic dysfunction, is not able to bear loads, and does not exhibit normal kinematics during any portion of the wrist’s arc of motion [1], [2]. Radiologically, instability traditionally has been defined in simple terms by a loss of normal bone alignment either at rest, termed static instability, or during active motion, termed dynamic instability [3], [4]. Although there are at least 8 major classification systems for carpal instability [5], the term “instability” implies that there is actual dysfunction in the normal kinetics (in which physiologic loads are transferred without sudden abnormal changes in stress upon the ligaments and cartilage) and the normal kinematics (in which the bones have a normal range of motion, without abnormal change in alignment dynamically during any portion of its arc of motion). While instability has been defined and classified in several ways, two of the most commonly types are scapholunate (SL) dissociation, and distal radioulnar joint (DRUJ) instability. In order to diagnose static and dynamic instability at these sites, numerous radiographic measurements have been proposed, including SL interval, SL angle, capitolunate (CL) angle, radiolunate (RL) angle and DRUJ subluxation ratio [3], [4]. Although these measurements are commonly applied to static examinations (i.e., with the wrist immobilized in the imaging system), radiography in multiple positions or with stress may be needed to diagnose instability [6]. Dynamic four-dimensional computed tomography (CT) has been employed to document the kinematics of the wrist bones during active motion [7]–[10] and coupled with CT arthrography has the potential to provide a diagnosis of intrinsic and extrinsic carpal ligament injuries [11]. Fluoroscopy also has been advocated as a supplementary technique for diagnosis of dynamic wrist instability [12]. Dynamic CT and fluoroscopy, however, involve additional ionizing radiation to the patient and provide poor contrast for soft tissue components of the wrist.

Magnetic resonance imaging (MRI) has the advantage of producing improved soft tissue contrast compared to other imaging modalities, without the concerns of ionizing radiation. Conventional non-contrast MRI studies of wrist instability performing static imaging have focused largely on the diagnosis of ligament derangements in the neutral position. These investigations however have reported inconsistent results. Using arthroscopic and arthrographic techniques as reference standards, studies have found that conventional MRI does not consistently diagnose all tears of the SL ligament, lunotriquetral ligament, or triangular fibrocartilage complex [13]–[15]. Arthrography and MR arthrography are often performed, but these exams can show asymptomatic ligament defects that may have minimal clinical (or biomechanical) significance, with attritional perforations beginning to appear in the third decade [16]. Thus, there is room for improvement in the current MR techniques used in the evaluation for wrist instability. Conventional MRI of the immobilized wrist may not yield the same information as that provided by a dynamic exam [17]. A dynamic exam has the additional potential to show any clunk or sudden change in intercarpal alignment.

Fast gradient-echo MRI pulse sequences termed “balanced steady-state free precession” (bSSFP) are capable of generating images rapidly (e.g., <600 ms per image) with a matrix size of 128×128, and with high ratios of both signal-to-noise and contrast-to-noise [18]. In a single-slice sequential acquisition mode, bSSFP has been applied successfully to dynamic imaging of the actively moving temporomandibular joint [19], but not to study static and dynamic instability in the wrist. There has been concern that bSSFP may not be technically feasible in the moving wrist because relatively small magnetic field inhomogeneities can generate substantial banding artifacts [18].

An MRI protocol, which we term “active-MRI”, was developed for evaluating the wrist during active motion. We use the “active-MRI” terminology here instead of “dynamic-MRI” to avoid potential confusion with dynamic (contrast-enhanced) MRI, which refers to scanning conducted to measure MRI contrast agent dynamics, or with “cine-MRI” where static MR images are played in a loop. Our objectives in this work were [i] to evaluate the technical feasibility and reliability of acquiring bSSFP images during active wrist motions (i.e., during active supination/pronation, radial/ulnar deviation, a dynamic clenched fist maneuver, and volarflexion/dorsiflexion), and [ii] to show that the resulting images enable the standardized derivation of metrics typically associated with wrist instability (i.e., DRUJ subluxation ratio, ECU tendon translation, SL interval, ulnar variance, and the SL, RL and CL angles).

## Materials and Methods

### Study Subjects

Ten asymptomatic volunteers (7 men, 3 women; average age 36 years; age range 27–58 years) were recruited for this prospective, HIPAA-compliant study. The study had approval from the University of California, Davis Institutional Review Board (IRB) and was conducted at the University of California, Davis, USA. Written informed consent was obtained for each volunteer based on approved IRB documentation prior to study initiation. Inclusion criteria for this study included asymptomatic wrists, age <60 years, and the ability to follow directions to perform wrist motions while in the MRI scanner. Exclusion criteria were contraindications to MRI (including claustrophobia) and history of wrist derangements (including trauma and arthritis).

### Imaging Protocol Development

**Figure 1:**
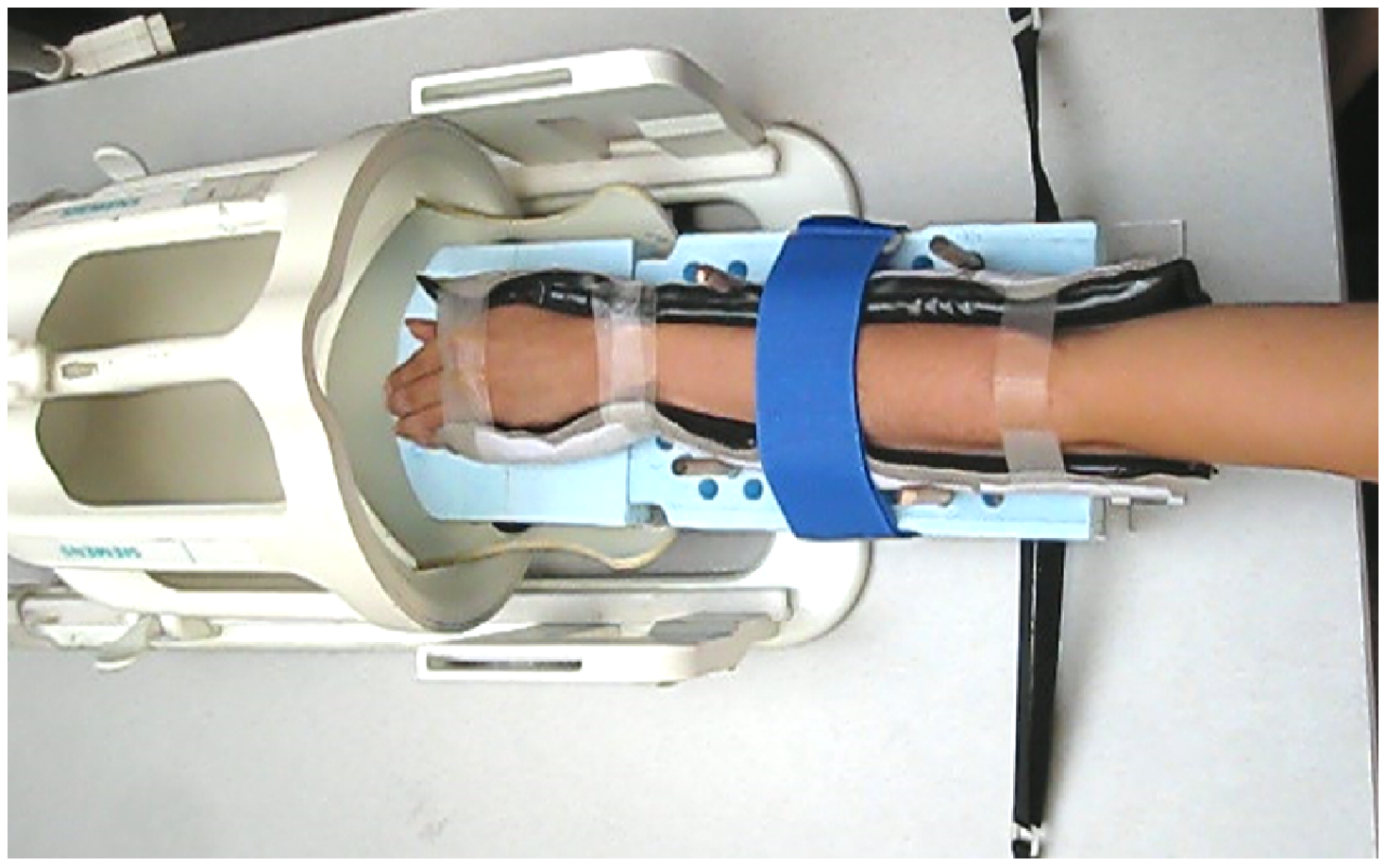
The forearm of a healthy volunteer in the wrist harness. The harness has been slid outwards for better visualization of both the harness and the MR coil.

MRI was performed on a 3-T system (VB17A Magnetom Trio, a Total Imaging Matrix System; Siemens Healthcare, Erlangen, Germany) equipped with an 8–channel radiofrequency (RF) head coil (Invivo Inc., Gainesville, FL, USA). Prior to commencing the study, initial imaging sessions were conducted in two healthy volunteers in order to develop the active-MRI protocol. The following three steps were undertaken. First, we confirmed that susceptibility artifacts, specifically banding artifacts that are unique to bSSFP sequences, partially obscured the wrist during motion. These artifacts were reliably minimized in the region of interest by using dielectric pads containing a perfluorocarbon liquid (Sat-Pad, Image Engineering Laboratories, Basking Ridge, NJ). In particular, these pads reduced magnetic field inhomogeneity across the volume of the wrist joint, and resulted in the banding artifact appearing only in the periphery of the imaged slice, outside the anatomic structures of interest. The pads eliminated the need for real-time dynamic shimming, which was not available on our MRI system. Pads were attached to the patient using medical grade tape so that they maintained contact with the wrist (**Figure 1**) but did not interfere with the range of motion (ROM), except for the volarflexion position in three of the early subjects in our study. For the radial/ulnar deviation maneuvers, these pads were attached to the medial and lateral sides of the wrist, while pads were attached dorsal and palmar to the wrist for the supination/pronation and volarflexion/dorsiflexion maneuvers.

Second, a positioning harness was designed to allow for the wrist to undergo maximal ROM along the principal axes (**Figure 1**). This harness, composed of an acrylic base with 3.2-cm-thick polystyrene foam, restrained transverse motion of the forearm through the use of dowels inserted into selected holes (selected from an array of holes in the foam, drilled to accommodate variable forearm widths) and Velcro straps to restrict forearm motion away from the tabletop in a vertical direction. The device also included a fastening mechanism of Velcro straps for attachment to both the coil and MRI table in a reproducible manner. The base was manufactured using 3-dimensional printing technology, and the digital design file is available upon request from the authors. The foam was machined on a workshop mill.

Third, imaging experiments were performed to determine pulse sequences and scanning parameters to provide satisfactory contrast resolution and a satisfactory trade-off between temporal and spatial resolution. Initial images acquired using the spin-echo echo-planar imaging pulse sequence had substantial spatial distortion and were unsuitable for this study, while images acquired using a single shot turbo spin echo sequence had an insufficient signal to noise ratio (SNR), due to single slice acquisition using 90° and 180° pulses and short TR in the 300–500 ms range). Gradient recalled echo (GRE) sequences provided images with significant tissue susceptibility-related signal loss, and also relatively low SNR, and were deemed unsatisfactory. The bSSFP sequence, referred to by the vendor as true fast imaging with steady state precession (true-FISP) provided very high SNR images with essentially no geometric distortion (since the sequence is spin-echo based). The true-FISP sequence was evaluated using 3-, 6-, and 10-mm section thicknesses. For each of the orthogonal slice orientations, optimal pulse sequence parameters were selected by consensus based on image review by four co-investigators (MHB, AJC, GJS, RDB), two of whom have greater than 10 years of experience in pulse sequence optimization.

The parameters for the true-FISP bSSFP sequence leading to acceptable spatial and temporal resolution converged to those providing 60 images with a 0.94 mm in plane spatial resolution, a 6 mm section thickness and 475 ms (coronal and axial orientations) or 562 ms (sagittal orientation) acquisition time per image (detailed sequence parameters are listed in **Table 1**). The combination of base resolution, phase resolution and phase partial Fourier on the MRI system led to an overall k-space matrix size of 128 in the frequency-encode direction, and 98 in the phase-encode direction. The bandwidth was selected to produce the shortest TR to reduce the possibility of the banding artifacts obscuring the anatomical structures of interest. The TE was equal to ½ TR in this balanced sequence. Centric acquisition ordering was used. Phase oversampling of 44%, effectively increasing the FOV in that direction to 172.8 mm, was used to improve SNR and eliminate any possibility of wrap-around artifact at the widest section of the hand and wrist. Compared to the parameters used to generate the axial and coronal images, a lower bandwidth, and consequently a longer TE, TR and scan time per image were used to generate the sagittal images, because by consensus higher SNR was preferred in the sagittal images. Further, it was decided that the subject would be instructed to move his/her wrist through no less than one cycle of the full range of wrist joint motion over approximately a 35 second period, thus providing 60 images over at least one pass of the full range of wrist joint motion.

**Table 1:** MRI sequence (true-FISP) parameters used for the study.

Parameter	Coronal/Axial	Sagittal
TR (repetition time)	3.98 ms	4.71 ms
TE(echo time)	1.99 ms	2.36 ms
Flip angle	47 degrees	47 degrees
Averages	1	1
Field of view	120 mm×120 mm	120 mm×120 mm
Section thickness	6 mm	6 mm
Base resolution	128	128
Phase resolution	100%	100%
Phase partial Fourier	4/8	4/8
Phase encode direction	R>>L/A >>P	A>>P
Phase oversampling	44%	44%
Acquisition ordering	Centric	Centric
Asymmetric Echo	Off	Off
Bandwidth	781 Hz/Px	454 Hz/Px
Parallel imaging	Off	Off
Scan time per image	475 ms	562 ms
Final image resolution	0.94 mm×0.94 mm	0.94 mm×0.94 mm

### Healthy Volunteer Scanning

Each volunteer was placed in the “superman position” (with one arm out-stretched above the head into the RF coil) while outside the scanner bore. The wrist, surrounded by dielectric pads that limit susceptibility artifacts, was placed into a wrist harness and each volunteer was trained to perform the following four wrist maneuvers, utilizing his/her full, active ROM (absent pain), at a comfortable speed, i.e., continuously between the “start” and “stop” instruction interval of 35 s: [i] radial/ulnar deviation; [ii] the clenched fist maneuver with the wrist in the neutral position and in ulnar deviation; [iii] supination/pronation; [iv] volarflexion/dorsiflexion. The volunteer was then moved into the scanner bore. Automated high-order B_0_ field shimming was performed on the wrist while it was motionless in the neutral position. This was followed by each of the maneuvers, with 2D image acquisitions in three planes. Five volunteers in the prospective study had bilateral exams performed successfully on a single MRI scanner during July 2012, and five additional subjects had a single wrist imaged. Each acquisition took ∼ 35 s. The time required for detaching and re-attaching the dielectric pads was ∼ 2 min. The entire exam took ∼10 min per wrist once the patient was positioned on the system. This time included shimming with the wrist in the neutral position, localization and slice selection.

### Image Analysis

**Figure 2:**
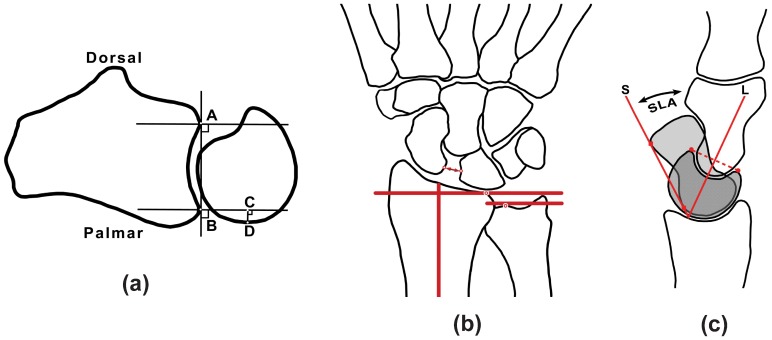
Metrics associated with wrist instability that were derived from standardized measurements on active-MR images. (a) **DRUJ subluxation ratio** – the subluxation ratio for the DRUJ was determined on axial images by connecting the palmar and dorsal aspects of the sigmoid notch and drawing perpendicular lines. The subluxation ratio was computed as a ratio between CD and AB; (b) **SL interval and ulnar variance** – the measurement of SL interval (two-sided arrow) and ulnar variance (distance between the dots with white centers on the solid red lines) was performed on the coronal images. The solid red lines represent the most distal aspect of the distal ulna and most proximal aspect of the distal radius articular surfaces; (c) **SL, RL and CL angles** – the axis of the scaphoid (S) was drawn by connecting the proximal and distal poles along the volar cortex (dotted line) on sagittal images. Similarly, the axis of the lunate was determined by connecting the distal dorsal and distal palmar corners and creating a perpendicular line. The SL angle (SLA) was determined by measuring the angle between the scaphoid and the lunate (L) axes. Similarly, the axes of the capitate and the radial shaft were determined and the CL angle was measured as the angle between the capitate and the lunate axes (not shown). The RL angle was measured as the angle between the long axis of the radial shaft and the lunate (not shown).

**Figure 3:**
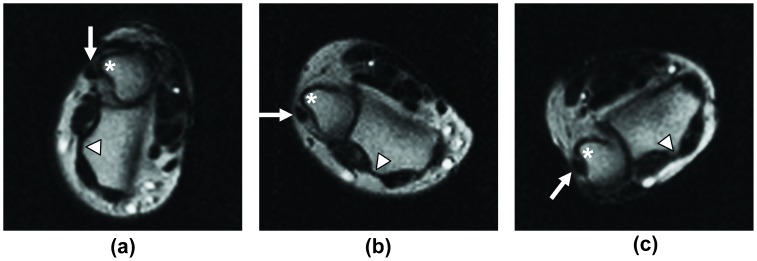
Extensor carpi ulnaris (ECU) tendon translation during wrist rotation. The relationship of the ECU tendon (arrow) to its groove as the forearm is rotated from (a) pronation, through (b) neutral to (c) supination – on axial images of the DRUJ using the active-MRI scan. In this volunteer, the ECU tendon was located within its groove in pronation, while in the neutral position, the tendon is subluxated eccentrically at the margin of the ulnar groove. In supination, the tendon is dislocated. Also visualized is the trajectory of the ulnar styloid process (white star) during the supination/pronation maneuver. Lister's tubercle (white triangle) at the dorsal aspect of the radius is shown as an anatomical reference point.

Measurements were performed by a blinded fellowship-trained musculoskeletal radiologist (RDB) and a blinded orthopedist (II) exclusively subspecializing in hand/wrist surgery, by consensus. All measurements were performed using the digital ruler and angle measurement tools available in the clinical viewing software (iSite PACS, Ver. 3.6, Philips Healthcare, Andover, MA). Specific anatomic measurements were performed in neutral and at the maximal endpoints of the ROM in each of the three planes as follows: [i] DRUJ congruity (**Figure 2a**) [20] and translation of the extensor carpi ulnaris (ECU) tendon on axial images in neutral, pronation and supination positions (**Figure 3**); [ii] the SL interval and ulnar variance on coronal images in neutral, clenched fist, radial and ulnar deviation positions (**Figure 2b**); and [iii] the SL, RL and CL angles on sagittal images in neutral, and dorsiflexion positions (**Figure 2c**).

On axial images, the DRUJ congruity was assessed using the “DRUJ subluxation ratio method” [20]. The ECU tendon translation was assessed at the same level by observing the position of the tendon within its groove. The tendon was considered “dislocated” if it was entirely outside its groove, and “subluxated” if it was partially contained in its groove. The relative locations of the ulnar styloid process and the Lister’s tubercle were used to determine whether the forearm was in neutral, supination, or pronation positions; the ulnar styloid process is normally on the dorsal side of the carpus in supination, and is located more palmarly in pronation [21], [22].

On coronal images, the SL interval was measured through the middle of the SL articulation. The distance between the cortices of the scaphoid and lunate was recorded, at the half-way point between the Gilula lines of the midcarpal and radiocarpal joints [23]. These measurements were repeated in neutral, ulnar deviation, and radial deviation wrist positions, as well as during the clenched fist maneuver. Ulnar variance was also assessed on the coronal images, by measuring the distance between the most distal aspect of the distal ulnar head cortex and the adjacent most proximal aspect of the distal radius cortex.

On sagittal images, the CL and RL angles were measured through the center of the lunate. A line was drawn connecting the palmar and dorsal corners of the lunate, and a line perpendicular to it was considered the lunate axis. The angles between lunate axis and the longitudinal axes of the capitate and radius were measured to establish the CL and RL angles, respectively. Sagittal images performed through both the scaphoid and the lunate during the volarflexion/dorsiflexion movement enabled the determination of the SL angle, according to the method modified from Maizlin and Vos [24]. The two sets of sagittal images were viewed side-by-side, and wrist flexion angles were matched by measuring the alignment of the metacarpals and the radial shaft. A single osseous landmark (the radial shaft) was used, and the angles between it and scaphoid axis (on one image) or lunate axis (on the other image) were determined. These angles were then added to calculate the SL angle.

## Results

### Active-MRI Protocol

**Figure 4:**
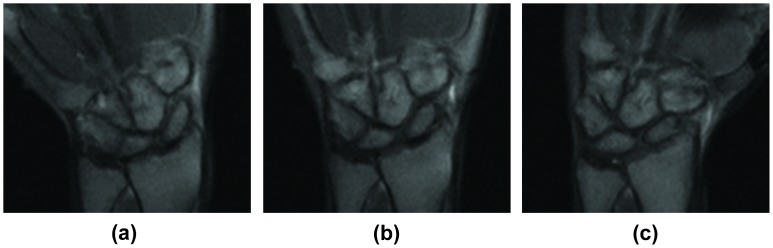
Active-MRI of the wrist during active ulnar-radial deviation. Snapshots of the coronal images of the wrist in the ulnar deviation (a), neutral (b) and radial deviation (c) positions during the continuous radial-ulnar deviation maneuver. SL interval and ulnar variance (see Figure 2 for these definitions) were measured from the resulting images.

Representative snapshots obtained from the active-MRI protocol are shown in **Figure 3** and **4**. Representative movies showing active-MR images acquired during the radial/ulnar deviation, pronation/supination, and volarflexion/dorsiflexion are available as **Movies S1, S2, and S3**. With a subject in position on the MRI system for wrist evaluation, the data acquisition time was <35 s for each maneuver.

### Quantitative Measures from Active-MRI

Detailed measurement results are recorded in **Table 2** **,** **3** **,** **4** **, and** **5**. Briefly, during wrist rotation from supination to neutral to pronation, the ulna tended to translate in the dorsal direction relative to the sigmoid notch of the radius, with a slight increase in the DRUJ subluxation ratio. The SL interval with a relaxed fist in both neutral and ulnar-deviated positions increased during the clenched fist maneuver. All volunteers performed the neutral and dorsiflexion positions of the volarflexion/dorsiflexion maneuver; however, due to physical restrictions of the scanning apparatus, a subset of the volunteers (3 volunteers, 5 wrists) were unable to freely move their wrists in volarflexion beyond neutral. For this reason, measurements are reported for neutral and maximal dorsiflexion positions only.

**Table 2:** Quantitative metrics derived from active-MRI images of the wrist during the pronation/supination maneuver.*

*Metric*		*Pronation*	*Neutral*	*Supination*
DRUJ subluxation ratio in dorsal direction [mean (range)] **		0.14 (0.06 to 0.27)	0.10 (0.06 to 0.2)	0.04 (−0.06 to 0.17)
ECU tendon location relative to its groove	Dislocated	1/14 (7%)	2/14 (14%)	6/14 (43%)
	Perched	2/14 (14%)	7/14 (50%)	5/14 (36%)

One volunteer unable to complete the pronation/supination motion protocol.

DRUJ subluxation ratio, as described by [20].

**Table 3:** Quantitative metrics derived from active-MRI images of the wrist during radial/ulnar deviation.

*Metric*	*Ulnar deviation*	*Neutral*	*Radial deviation*
SL gap (mm) [mean (range)]	1.43 (1 to 2)	1.43 (1 to 2)	1.43 (1 to 2)
Ulnar variance (mm) [mean (range)]	−0.93 (0 to −2)	−0.93 (0 to −2)	−0.92 (0 to −2)

**Table 4:** Quantitative metrics derived from active-MRI images of the wrist during the clenched fist maneuver.

*Metric*	*Relaxed fist*	*Clenched fist*
SL gap (mm) [mean (range)]	1.36 (1 to 2)	1.64 (1 to 3)

**Table 5:** Quantitative metrics derived from active-MRI images of the wrist during the neutral and dorsiflexion.*

*Metric*	*Neutral*	*Maximum dorsiflexion*
RL angle (°) in dorsal direction [mean (range)]	3.7 (0 to 17)	29.5 (12 to 49)
CL angle (°) in dorsal direction [mean (range)]	4.9 (−8 to 17)	26.7 (7 to 46)
SL angle (°)[mean (range)]	59 (34 to 84)	48.5 (29 to 69)

The wrist harness limited the ability for achieving the full range of volarflexion in a subset of volunteers therefore measurements for volarflexion are not reported.

## Discussion

Wrist instability is a complex phenomenon that has been studied extensively. There are potentially discordant findings during static and dynamic examinations, and there is substantial interest in diagnostic imaging performed during motion or loading [25]. MRI of the static wrist has been performed in various positions [26] and with stress [27], but we are not aware of peer-reviewed publications showing the feasibility of *in vivo* real-time MRI during active, full ROM of the wrist.

The bSSFP family of fast gradient echo pulse sequence is best known for their application in real-time dynamic cardiovascular imaging. Vendor-specific names for bSSFP pulse sequences include true Fast Imaging with Steady Precession (true-FISP, Siemens), Fast Imaging Employing Steady sTate Acquisition (FIESTA, General Electric), and balanced Fast Field Echo (bFFE, Philips). Tissue image intensity is determined by each tissue's T_2_/T_1_ ratio, and consequently fat and fluid usually appear with greater image intensity, i.e. brighter, than other tissues [28]. The bSSFP sequences use an ultrashort TR (typically in the range of 3–6 ms, allowing for fast k-space acquisition with sufficiently large matrix sizes, e.g., 128 or 256 in each dimension). Due to use of alternating polarity RF pulses, bSSFP sequences have large steady-state magnetization, which provides a high SNR [19]. In the present study, we found that wrist images can be acquired rapidly (<35 s) during motion in a reliable manner using a bSSFP pulse sequence, particularly when artifacts are minimized by the use of dielectric pads. Our overall aim was to develop an active-MRI protocol to be a short supplement to a conventional MRI exam that could potentially augment high spatial resolution images of the latter with high temporal resolution images of the former, but not to compete with or replace the latter.

Active-MRI enabled the measurement of metrics typically associated with dynamic wrist instability in all subjects, including those associated with some soft tissue components like the tendons. The measurements obtained in our study are in agreement with values reported for normal subjects as we describe below. The ECU tendon had greatest displacement from its groove in supination, followed by neutral, and was least displaced in the pronated position as has been reported earlier [29]. Similar to our results, a study of asymptomatic wrists detected a 58% rate of ECU subluxation, half of which were completely displaced from the groove [30]. The reported normal values for the DRUJ subluxation ratio [20] are similar to what we obtained in the current study, with increasing (relative) dorsal subluxation as the wrist was moved from supination into pronation. Reported normal SL, RL, and CL angles range between 30° to 80°, −30° to 30°, and −10° to 30°, respectively, depending on the radiographic study [31]. Our measurements obtained in the neutral position are within the range of normal radiographic values. With wrist extension, there is complex motion of wrist bones in multiple planes, with the lunate, capitate, and scaphoid demonstrating increased angulation dorsally; the scapholunate motion has been reported to be 19° during extension, with a resulting decrease in the SL angle [32]. Our results are in line with these findings, as we also demonstrated a decrease in the SL angle, along with increases in both the CL and RL angles, in asymptomatic volunteers. Similarly, ulnar variance measured in our study using active-MRI was well within the range reported for normal subjects using radiography [33]. Finally, we did not see any abnormalities in the SL interval in our study of asymptomatic volunteers. A commonly-accepted threshold of normal SL interval is less than 3 mm, and none of our subjects displayed instability. Further work with symptomatic patients known to have SL instability will be needed to confirm the validity of this technique.

A limitation of the proposed active-MRI protocol is that the spatial resolution had been traded-off for temporal resolution. We are exploring three approaches to improve upon the spatial and temporal resolution obtainable with bSSFP. First, we are exploring the use of a higher peak gradient strength, since this would allow higher spatial resolution to be achieved without compromising acquisition time or the SNR. Currently, the manufacturer imposes 27 mT/m peak gradient strength in all of its commercial pulse sequences, yet the rated and advertised maximum for orthogonal imaging is 40 mT/m. Second, an approach to increasing spatial resolution, without increasing acquisition time, is the use of parallel imaging in one or two dimensions, or the use multi-band acquisition. A multi-channel RF coil custom-fitted for the wrist would be needed for such techniques, replacing the large 8-channel head coil. Third, the current true-FISP sequence allows acquiring 2D images only at a single slice location. 3D data acquisition with true-FISP [34], with or without multidimensional parallel imaging, is desirable for dynamic wrist imaging. We are investigating modification of the true-FISP pulse sequence to allow 3D data acquisition, and also interleaved multi-phase, multi-slice 2D slice acquisitions (i.e., acquisition of tissue volumes in a time-series using multi-slice 2D acquisition, as done in functional MRI of the brain with 2D echo planar imaging).

Our study had limitations. In order to facilitate standardized, reproducible measurements in the axial, coronal and sagittal planes, we constructed a harness to constrain wrist motion in the typical orthogonal planes during active motion. Unfortunately, this custom-designed harness presented a mechanical obstruction that limited full range of volarflexion in three of our early subjects, and therefore we do not report metrics for this position. As we learned with experience, full ROM is not difficult to achieve when the wrist was positioned to allow movement freely within the coil. For this study, we used a head coil to allow space for the performance of the maneuvers during scanning. This choice led to a poorer SNR compared to dedicated wrist coils. We are exploring designs of flexible wrist coils that could be used for this purpose in the future.

Another limitation of this study is that it is only a “proof of concept” investigation carried out in a small number of asymptomatic volunteers, without any evaluation of symptomatic patients or comparison to a reference standard. Additional studies are necessary to establish the usefulness active-MRI in patients with suspected dynamic wrist instability. Further, correlative studies of active-MRI with arthroscopic or surgical findings [2], [11], [35] are also needed in order to determine the potential role of active-MRI in the management of dynamic wrist instability. Finally, since active-MRI lacks the spatial resolution typically afforded by high resolution static MRI, especially for the interrogation of the components of the main intrinsic and extrinsic carpal ligaments, work is necessary to determine an optimal way to synthesize such fast images with those from routine static MRI when examining patients clinically for dynamic wrist instability.

The focus of our investigation was on active-MRI, however, there are other imaging techniques that ultimately may prove clinically useful. Of particular recent interest is four-dimensional CT (4D-CT), in which high temporal resolution images can be acquired during wrist motion on the latest generation of CT scanners such as multirow detector CT (MDCT) [36] systems. The dynamic CT technique has been reported in a cadaveric wrist [10], in two healthy volunteers [37], and in a cohort of 4 patients with one volunteer [9]. A limitation of CT is the additional ionizing radiation to the patient for each dynamic maneuver over and above a standard static acquisition in the neutral position.

## Conclusion

This pilot study documents the development of a protocol for active-MRI using a bSSFP pulse sequence that reliably provides real-time image acquisition during movements and consequently, enables measurements of acceptable anatomical parameters associated with dynamic wrist instability. Given the short acquisition time (<35 s), additional work is warranted to assess active-MRI that is optimized for temporal resolution as a possible supplement to conventional static images acquired during routine MRI exams that are typically optimized spatial and contrast resolution. Additional studies are also needed for correlating active-MRI metrics with arthroscopic or surgical findings of dynamic wrist instability, and with techniques such as MR arthrography and dynamic CT.

## Supporting Information

Movie S1 **Active-MRI during radial/ulnar deviation.**
(AVI)

Movie S2 **Active-MRI during the pronation/supination maneuver.**
(AVI)

Movie S3 **Active-MRI during the neutral/dorsiflexion maneuver.**
(AVI)
